# The link between cognitive abilities and risk preference depends on measurement

**DOI:** 10.1038/s41598-023-47844-9

**Published:** 2023-11-30

**Authors:** Sebastian Olschewski, Jörg Rieskamp, Ralph Hertwig

**Affiliations:** 1 Department of Psychology, Center for Economic Psychology, University of Basel, Missionsstrasse 62A, 4056 Basel, Switzerland; 2 Warwick Business School, University of Warwick, Coventry, UK; 3 Max Planck Institute for Human Development, Berlin, Germany

**Keywords:** Human behaviour, Cognitive neuroscience

## Abstract

Risk preference is an important construct for understanding individual differences in risk taking throughout the behavioral sciences. An active stream of research has focused on better understanding risk preference through its connection to other psychological constructs, in particular, cognitive abilities. Here, we examine two large-scale multimethod data sets and demonstrate that the method used to measure risk preference is an important moderator. In self-report measures, we found small but consistent positive correlations between working memory capacity/numeracy, facets of cognitive abilities, and risk tolerance. In behavioral measures, we found, on average, no correlation and large intermethod heterogeneity. This heterogeneity can be explained by the choice architecture that is created in behavioral methods—in particular, the relation between risk and reward and the impact of decision error in a task. Consequently, investigating how risk preference relates to psychological constructs such as cognitive abilities require a profound understanding of the choice architecture in measurements of risk preference and in the real world.

## Introduction

Risk preference is an important psychological construct to explain behavior in many everyday situations where people face risk and uncertainty. Examples are career choices, engaging in risky recreational activities, or investing in the stock market. Importantly, people differ in their propensity to take risks in these choices, and behavioral scientists strive to explain these differences in behavior by focusing on respective differences in people’s risk preferences. To fully explain difference in choices, risk preferences have also been linked to differences in cognitive abilities. Cognitive abilities encompass different dimensions of intelligence such as fluid and crystallized intelligence, executive function, and working memory capacity^1–3^. For example, higher cognitive abilities correlate with educational attainment, employment opportunities, and income^4,5^. Moreover, higher cognitive abilities relate to higher stock market participation, even when controlling for income and wealth effects^6–8^. Historically, stock market investments have involved higher risks but have also yielded superior long-term returns relative to less risky investment options such as bonds or savings accounts^9–11^. Through this link, higher cognitive abilities could be positively related to people’s long-term wealth.

Here, we examine whether cognitive abilities, measured with working memory capacity and numeracy, are related to risk preference across a large number of risk preference elicitation methods. Working memory capacity is an established construct that is correlated with, but not identical to, general intelligence^12–16^. As part of the executive functions, working memory capacity is related to many aspects of life including mental health, educational achievements, and job success^17^. Numeracy describes the ability to work with numeric information and has been shown to influence decision making involving processing probabilities and numbers as required for risky choices^18–21^. Like working memory capacity, numeracy is correlated with, but not identical to, fluid intelligence^22^.There are various accounts postulating how a positive relation between cognitive abilities and therefore also working memory capacity/numeracy and risk tolerance (Table 1) could emerge. Some propose direct cognitive or affective reasons why people with higher cognitive abilities accept more risks^23,24^; others assume more indirect environmental or demographic factors^25,26^.

**Table 1:** Explanations for a positive relation between cognitive abilities and risk tolerance.

Citation	Explanation
Frederick^23^	Riskless or low-risk options are intuitively more attractive. Cognitive effort is required to overcome this attraction and choose riskier options
Read et al.^24^	Lower cognitive abilities lead to narrower choice bracketing, which makes risks more aversive than if choices are construed as assuming a wide (e.g., temporal) context
Dohmen et al.^27^; Frey et al.^25^	Cognitive abilities and risk tolerance coevolve in the environment. For example, riskier education choices are more likely to be rewarded if cognitive abilities are high
Arslan et al.^28^; Bugg et al.^29^; Josef et al.^30^; van den Bos & Hertwig^31^	Age is negatively related to (fluid) cognitive abilities and risk tolerance. Age thus mediates the relation between cognitive abilities and risk tolerance

The empirical evidence for a positive relation between cognitive abilities and risk tolerance is mixed. Possible reasons are that there is neither a universally accepted definition of risk nor a clear understanding of how risk is perceived^32–34^. Consequently, there is no consensus on which elicitation method for risk preference is best suited to examining the relation between cognitive abilities and risk preference. Generally, there are two distinct measurement traditions: self-report questionnaires and elicited behavior^35,36^. Self-report measures ask for a self-assessment of past or hypothetical behavior; behavioral measures ask for (usually small-stake and incentive-compatible) choices between different risky options. Importantly, correlations between these different elicitation methods are weak^37^.

The link between cognitive abilities and risk preference has mainly been investigated using behavioral measures. Commonly, these studies examined only one or two behavioral measures. A recent meta-analysis concluded that the correlation between cognitive abilities and risk tolerance, elicited through behavioral measures, is positive but weak^38^. Another recent meta-analysis, focusing on one specific elicitation format (multiple price lists), found no support for this correlation^39^. A few studies have examined both self-report and behavioral measures (e.g.,^40^). Of these, one observed a positive correlation between cognitive abilities and risk tolerance across both self-report and behavioral methods^25^. Another found that cognitive abilities were related to behavioral but not to self-report risk preference measures^41^. A third study observed no notable relation in either elicitation method after controlling for other individual differences such as age^26^.

These diverse empirical findings suggest the existence of moderators for the association between cognitive abilities and risk preference. We propose that a key moderator is the elicitation method—in particular, the method’s choice architecture. When designing a behavioral elicitation method, researchers construct a choice architecture with a specific range of possible outcomes and probabilities that define the risk and return of the choice options^42^. These features of the choice architecture lead to method-specific relationships between risks and rewards^43^. Consequently, people’s observed behavior can, at least partly, be the result of an elicitation method’s specific choice architecture and not or not exclusively their risk preference. Choice architecture and risk–return relationships in elicitation methods are usually not controlled for across methods and thus may represent a key source of heterogeneity in empirical findings. Next, we present different ways through which a positive correlation between higher cognitive abilities and risk tolerance manifests spuriously.

Assume a choice architecture consisting of two choice situations 1 and 2 (see Table 2). In each pair of lotteries, the riskier lottery A (with the two possible outcomes being further apart) always has a higher expected value (EV) than lottery B. Someone who consistently maximized EV would choose the risky lottery in both situations and would appear risk tolerant. If the same EV maximizer faced a choice architecture consisting of choice situations 3 and 4, in which the safer lotteries have a higher EV than the riskier ones, they would choose the safer option. The same person and choice strategy would therefore appear more risk averse in this environment than they would in the other if risk preference is measured by whether the riskier option is chosen. Although these examples are simplifications of more complex choice architectures of various elicitation methods, the logic remains the same. If an elicitation method entails a positive correlation between the riskier lottery and EV, then an EV maximizer could appear risk tolerant, but in cases of a negative correlation, risk averse.

**Table 2:** Example of a choice architecture and its effect on inferred preferences.

Choice situation	Lottery A	Lottery B	Risk-averse individual	Risk-tolerant individual	EV maximizer	Noisy EV maximizer
1	$85 with 50% probability, otherwise $20	$60 with 50% probability, otherwise $40	B	A	A	B
2	$90 with 50% probability, otherwise $35	$80 with 50% probability, otherwise $40	B	A	A	A
3	$80 with 50% probability, otherwise $20	$65 with 50% probability, otherwise $40	B	A	B	A
4	$90 with 50% probability, otherwise $30	$80 with 50% probability, otherwise $45	B	A	B	B

EV = Expected value.

Choice architecture is highly relevant to the question of whether there is a link between cognitive abilities and risk preference. There is evidence that people with higher cognitive abilities are more likely to maximize EV^44–47^. This could be because people with higher cognitive abilities are more likely to apply the goal of maximizing EV to a choice situation, or because they are less noisy in doing so (similar to findings in reasoning tasks^48^). Consequently, a positive relation between cognitive abilities and risk tolerance in a behavioral measure could be spurious and dependent on the correlation between risky and EV-maximizing options in a given choice architecture^27^. More generally, variation in the relation between EV-maximizing and risky lotteries across experimental tasks could explain the heterogeneity of the reported relations between cognitive abilities and risk preference in behavioral tasks.

Another feature of human decision making that interacts with a given choice environment is decision error. Decision error can be a result of cognitive imprecision in the processing of numeric information^49^ or in the execution of a decision. Returning to Table 2, consider a noisy EV maximizer who erroneously chooses an option that does not maximize EV. In choice situations 1 and 2, this noisy EV maximizer would appear more risk averse than an EV maximizer without error—but in choice situations 3 and 4, they would appear more risk tolerant. Although choice architectures are usually more complex, the same logic applies: If, in a given choice architecture, EV-maximizing and riskier lotteries are positively (negatively) correlated, then an increase in the error rate can make a person appear more risk averse (tolerant)^50,51^. In particular, a positive correlation between risks and returns is often applied in choice architectures to obtain a higher resolution in detecting different degrees of risk aversion.

This interaction between choice architecture and decision error is highly relevant to the link between cognitive abilities and risk preference because people with lower cognitive abilities could plausibly make more erroneous decisions^52–55^. However, inferring a positive correlation between cognitive abilities and risk tolerance on the basis of such an analysis would not be universal. Rather, it would only hold in a choice architecture in which risky options are more attractive to the majority of participants for other reasons (e.g., because of a higher EV).

Unlike behavioral elicitation methods, self-report measures appear less prone to introducing sources of spurious correlation between cognitive abilities and risk preference. Relative to behavioral measures they are less likely to impose a novel and situation-specific choice architecture. Rather, they tap into a person’s diagnostic past experiences and consequential behaviors in response to existing real-world choice architectures^28^. Thus, if there is a genuine link between cognitive abilities and risk preference, we expect a higher and more homogenous correlation for various self-report than behavioral measures. However, when explicitly asking about different domains as the DOSPERT scale^56^ does (e.g., financial, health, or social), self-report questionnaires could also relate to distinct sets of past experience, potentially leading to different relations between cognitive abilities and risk preference.

In sum, our research question is *if* and *how* working memory capacity and numeracy, as facets of cognitive abilities, are associated with differences in risk preference and whether this relation is similar across various elicitation methods of risk preference. The link between working memory capacity/numeracy and risk preference could be moderated by the elicitation method (self-report vs. behavioral) and the choice architecture especially within the behavioral elicitation methods. In this case, it is important to understand the exact path through which working memory capacity/numeracy is (sometimes) coupled with risk preference—and whether the coupling is real or a methodological artefact. To examine this issue, we used two large-scale data sets that employed a great variety of risk preference measures: one from Frey et al.^37^ (Data F) and one from Eisenberg et al.^57^ (Data E). Frey et al. assessed 1,507 participants, measured working memory capacity and numeracy, and examined 36 measures of risk preference. Eisenberg et al. assessed 522 participants, measured working memory capacity, and examined 12 measures of risk preference. All measures of risk preference are summarized in Table 3.

**Table 3:** Short descriptions of methods to elicit risk preference.

Abbreviation	Full name	Data set	Description	RR
*Self-report methods*				
GABS	Gambling attitudes and beliefs survey	Data F	Participants state their degree of agreement with 35 statements about gambling-related attitudes and beliefs	NA
SOEP	Socio-economic panel, general risk	Data F	Single question on whether a person is generally risk tolerant or risk averse	NA
SOEP_dri_	Socio-economic panel, driving	Data F	Single question on traffic-related risk preference	NA
SOEP_fin_	Socio-economic panel, financial	Data F	Single question on financial risk preference	NA
SOEP_rec_	Socio-economic panel, recreational	Data F	Single question on recreational risk preference	NA
SOEP_occ_	Socio-economic panel, occupational	Data F	Single question on occupational risk preference	NA
SOEP_hea_	Socio-economic panel, health	Data F	Single question on health-related risk preference	NA
SOEP_soc_	Socio-economic panel, social	Data F	Single question on social risk preference	NA
D_eth_	DOSPERT, ethics	Data F	Questions on likelihood of engaging in several behaviors in the ethics domain	NA
D_inv/fin_	DOSPERT, investment/financial	Data F, Data E	Questions on likelihood of engaging in several behaviors in the investment domain (part of financial domain)	NA
D_gam_	DOSPERT, gambling	Data F	Questions on likelihood of engaging in several behaviors in the gambling domain (part of financial domain)	NA
D_hea_	DOSPERT, health	Data F, Data E	Questions on likelihood of engaging in several behaviors in the health/safety domain	NA
D_rec_	DOSPERT, recreational	Data F, Data E	Questions on likelihood of engaging in several behaviors in the recreational domain	NA
D_soc_	DOSPERT, social	Data F, Data E	Questions on likelihood of engaging in several behaviors in the social domain	NA
PRI	Personal risk inventory	Data F	Choice between a riskier and a safer action in 13 hypothetical everyday scenarios	NA
BIS	Barratt impulsivity scale	Data F, Data E	30 self-assessment questions grouped into six factors: attention, cognitive stability, motor, perseverance, self-control, and cognitive complexity (only attention, motor, and self-control were used)	NA
SSSV	Sensation-seeking scale-V	Data F, Data E	40 questions where participants select one of two scenarios. Four factors: boredom, disinhibition, experience-seeking, and adventure-seeking	NA
*Behavioral methods*				
Lottery	Binary lottery choice task	Data F	Choosing 84 times between two fully described lotteries that differ in variance (adaptive design)	–0.02
MPL	Multiple price list	Data F	Sorted lists of several choices between two described lotteries that differ in variance	0.26
H&L	Holt and Laury^58^ multiple price list	Data E	Sorted list of 10 choices between two described lotteries that differ in variance	0.85
CCT-cold	Columbia card task, cold	Data F, Data E	Participants decide in advance how many of a set of 32 winning and losing cards to turn over	–0.25, –0.63
CCT-hot	Columbia card task, hot	Data E	Like CCT-cold, but participants decide sequentially whether to turn another card	0.95
MT	Marble task	Data F	Participants choose repeatedly between grids containing black and white marbles symbolizing wins and losses	NA
Traffic	Vienna risk-taking test—traffic	Data F	24 videos of situations where participants indicate the point at which they would no longer perform a maneuver that becomes riskier over time	NA
BART	Balloon analogue risk task	Data F	30 trials in which participants earn points by pumping up a balloon. If the balloon bursts, they lose all points	0.70
DfE	Decisions from experience	Data F	Eight trials in which participants sample freely from two options and choose one for an additional draw	–0.02
DfD	Decisions from description	Data F	Eight trials that were matched to DfE, but where all outcomes and their probabilities are described	0.00
Angling-k	Angling risk task, keep	Data E	30 trials in which participants earned a point for each fish they caught but lost all points if they caught a blue fish. Caught fish were not returned to the pond	0.55
Angling-r	Angling risk task, return	Data E	Like Angling-k, but caught fish were returned to the pond, keeping the risk the same for every round	0.58

*Note.* The relation between risk and return (RR) as a feature of a method’s choice architecture can be calculated for only some of the behavioral methods. In description-based methods, it refers to the correlation between choosing the riskier of two options and choosing the option that maximizes expected value (EV). In experience-based methods it is calculated as the empirical correlation between the number of risky choices and EV-maximizing choices or overall payoff. For more details concerning the tasks and stimuli, see Frey et al.^37^ for Data F and Eisenberg et al.^57^ for Data E.

## Results

### Are working memory capacity/numeracy and risk preference correlated?

**Figure 1:**
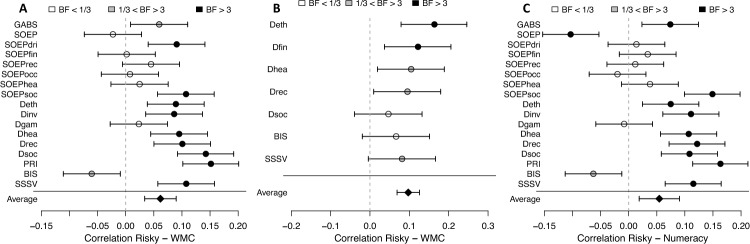
Self-report methods of risk preference elicitation and their correlation with working memory capacity and numeracy. Panel A: Data F correlation between working memory capacity and risk preference from self-report measures. Panel B: Data E correlation between working memory capacity and risk preference from self-report measures. Panel C: Data F correlation between numeracy and risk preference from self-report measures. Dot shading represents Bayes factors. Error bars indicate 95% frequentist confidence intervals. BF = Bayes factor as evidence for a correlation (H1) over evidence against a correlation (H0); WMC = working memory capacity. See Table 3 for abbreviations of the self-report methods.

Averaged across all self-report methods, the correlation between working memory capacity and risk tolerance was *M*_*r*_ = 0.06, *Med*_*r*_ = 0.09 (Data F) and *M*_*r*_ = 0.10, *Med*_*r*_ = 0.10 (Data E; see Fig. 1). This is a significant and credible correlation, according to both frequentist tests, Data F: *t*(16) = 4.33, *p* < 0.001; *W*(*n* = 17) = 142, *p* < 0.001; Data E: *t*(6) = 6.71, *p* < 0.001; *W*(*n* = 7) = 62, *p* = 0.016, and the Bayes factor, Data F: *BF*_1,0_ = 68; Data E: *BF*_1,0_ = 68, which was calculated by comparing the correlations of all individual measures against the null hypothesis of no correlation in both studies. Similar correlations are observed with numeracy, *M*_*r*_ = 0.05; *Med*_*r*_ = 0.07; *t*(16) = 2.99, *p* = 0.009; *W*(*n* = 17) = 131, *p* = 0.008; *BF*_1,0_ = 6. This means that participants with higher working memory capacity/numeracy reported higher risk tolerance, on average, across all self-report methods. However, the effect size of this correlation is small. A little more than half of the individual methods had a significant correlation with working memory capacity/numeracy, whereas for several methods in both studies, the Bayes factor suggested there was no relation. Among these methods were several from the Socio-Economic Panel, frequently used in prior studies on the relation between cognitive abilities and risk preference e.g., ^25^.

**Figure 2:**
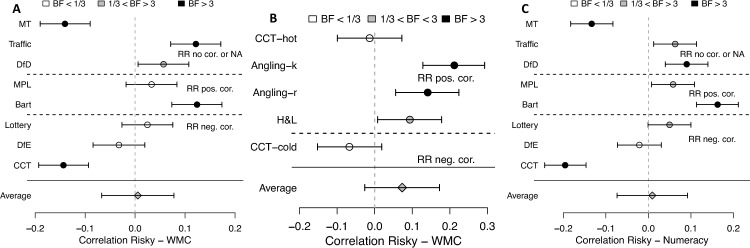
Behavioral methods of risk preference elicitation and their correlation with working memory capacity and numeracy. Panel A: Data F correlation between working memory capacity and risk preference from behavioral measures. Panel B: Data E correlation between working memory capacity and risk preference from behavioral measures. Panel C: Data F correlation between numeracy and risk preference from behavioral measures. Dot shading represents Bayes factors. Error bars indicate 95% frequentist confidence intervals. RR (relation between risk and return) refers to the characteristics of the choice architecture as explained in Table 3. BF = Bayes factor as evidence for a correlation (H1) over evidence against a correlation (H0); WMC = working memory capacity; NA = no correlation could be calculated. See Table 3 for abbreviations of the behavioral methods.

Averaged across all behavioral elicitation methods, the average correlation with working memory capacity was *M*_*r*_ = 0.01, *Med*_*r*_ = 0.03 (Data F) and *M*_*r*_ = 0.07, *Med*_*r*_ = 0.09 (Data E; see Fig. 2). That is, no consistent link between working memory capacity and risk preference emerged in the behavioral methods, Data F: *t*(7) = 0.15, *p* = 0.886; *W*(*n* = 8) = 19, *p* = 0.945; *BF*_1,0_ = 0.34; Data E: *t*(4) = 1.44, *p* = 0.223; *W*(*n* = 5) = 12, *p* = 0.312; *BF*_1,0_ = 0.79. However, there was enormous heterogeneity within these methods: Four methods (Vienna Risk-Taking Test—Traffic and the Balloon Analogue Risk Task in Data F and both Angling Risk Tasks in Data E) resulted in a significantly positive correlation between working memory capacity and risk tolerance, and two methods (the Columbia Card Task and the marble task in Data F) resulted in a significantly negative correlation. Importantly, in two of the most used risk preference elicitation methods—the binary lottery choice task and the multiple price list—there was Bayesian support for no correlation between working memory capacity and risk preference (Data F) or unclear evidence (Data E). A similar picture emerged for numeracy in Data F, *M*_*r*_ = 0.01; *Med*_*r*_ = 0.05; *t*(7) = 0.22, *p* = 0.836; *W*(*n* = 8) = 21, *p* = 0.742; *BF*_1,0_ = 0.34.

In all, we observed a small but consistent positive correlation between working memory capacity/numeracy and risk tolerance in the self-report methods, but no support for such an overall positive correlation in the behavioral methods. This suggests that the relation critically depends on how risk preference is measured. Within the behavioral methods, we observed enormous heterogeneity in the correlations. We now turn to the role of the choice architecture in the behavioral measures to shed light on the reasons underlying some of this heterogeneity.

### Does EV maximization explain the heterogeneity in the behavioral methods?

**Figure 3:**
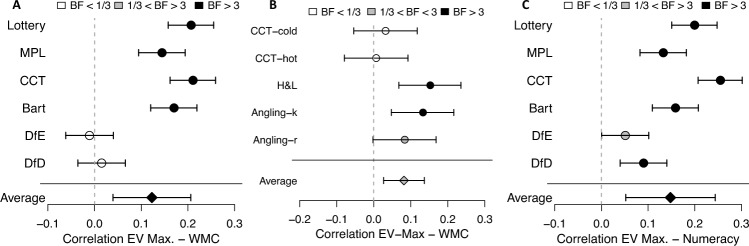
Behavioral measures of risk preference and correlation between EV-maximizing behavior and working memory capacity and numeracy. Panel A: Data F correlation between working memory capacity and expected value (EV) maximizing from behavioral measures. Panel B: Data E correlation between working memory capacity and EV maximizing from behavioral measures. Panel C: Data F correlation between numeracy and EV maximizing from behavioral measures. Dot shading represents Bayes factors. Error bars indicate 95% frequentist confidence intervals. BF = Bayes factor as evidence for a correlation (H1) over evidence against a correlation (H0); EV Max. = expected value maximization; WMC = working memory capacity. See Table 3 for abbreviations of the behavioral methods.

In all but two of the examined behavioral methods, we can determine the degree to which participants chose as if they maximized EV. EV maximization, unlike expected utility maximization, represents a risk-neutral preference. It is a choice strategy that requires some cognitive effort (i.e., determining a weighted sum, by multiplying monetary outcomes with their outcome probabilities and summing the products). We therefore hypothesized that this strategy would be more frequently or more consistently employed by people with higher (vs. lower) cognitive abilities. Importantly, the behavioral elicitation methods differ in their choice architecture and in the extent to which EV maximizing behavior means to more frequently choose safer or riskier options. In the two data sets, on average, working memory capacity was indeed positively correlated with EV maximizing (*M*_*r*_ = 0.13, *Med*_*r*_ = 0.16 for Data F; and *M*_*r*_ = 0.08, *Med*_r_ = 0.08 for Data E; see Fig. 3). This correlation was significant according to a *t* test in both data sets and had support according to the Bayes factor in Data F, Data F: *t*(5) = 3.10, *p* = 0.027; *W*(*n* = 6) = 20, *p* = 0.063; *BF*_1,0_ = 3.31; Data E: *t*(4) = 2.90, *p* = 0.044; *W*(*n* = 5) = 15, *p* = 0.062; *BF*_1,0_ = 2.44. The most robust correlation was observed between EV maximizing and numeracy, *M*_*r*_ = 0.15; *Med*_*r*_ = 0.15; *t*(5) = 4.92, *p* = 0.004; *W*(*n* = 6) = 21, *p* = 0.031; *BF*_1,0_ = 13.

To understand how the correlation between working memory capacity and EV maximizing can explain the heterogeneity in the link between working memory capacity/numeracy and risk preference, it is instructive to examine the choice architecture with some examples (Table 3): In the Columbia Card Task in Data F, EV-maximizing was tantamount to taking less risk. This might explain the negative relation between working memory capacity/numeracy and risk tolerance found for this method in Data F. In the Balloon Analogue Risk Task and both Angling Risk Tasks, in contrast, EV maximizing was tantamount to taking more risk. This could explain why positive correlations between working memory capacity/numeracy and risk tolerance emerged in these methods.

### Response errors

Choice architecture can also affect the estimates of the link between working memory capacity and risk preference through participants’ response errors. One way of examining this in behavioral methods is to look at the consistency of choices with respect to a latent utility order, which makes it possible to measure decision error within one session. Using a random utility model, we examined the latent choice consistency parameter in the binary lottery choice task and the multiple price list in Data F and the Holt and Laury multiple price list^58^ in Data E (see Supplemental Information). These consistency parameter estimates were strongly negatively correlated with working memory capacity and numeracy (*r*($\uptheta$_Lottery_, *WMC*) = –0.17, *p* < 0.001, *BF*_1,0_ = 5 × 10^7^; *r*($\uptheta$_Lottery_, Numeracy) = –0.18, *p* < 0.001, *BF*_1,0_ = 1 × 10^10^; *r*($\uptheta$_MPL_, *WMC*) = –0.25, *p* < 0.001, *BF*_1,0_ = 4 × 10^18^; *r*($\uptheta$_MPL_, Numeracy) = –0.23, *p* < 0.001, *BF*_1,0_ = 1 × 10^17^; and *r*($\uptheta$_HL_, *WMC*) = –0.25, *p* < 0.001, *BF*_1,0_ = 2 × 10^6^), meaning that participants with higher working memory capacity/ numeracy made more consistent choices with respect to the best-fitting latent utility order.

**Figure 4:**
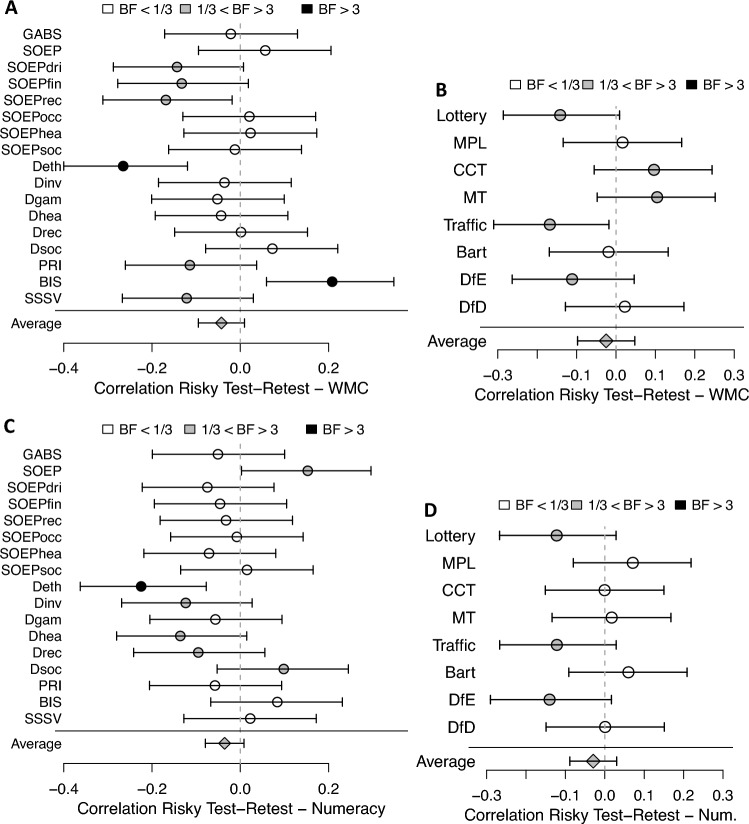
Correlation between absolute difference in risk preference measures at two measurement time points and working memory capacity and numeracy. Test–retest reliability measures of risk preference based on a subset of 171 participants from Data F. Panel A: Self-reported elicitation methods of risk preference and working memory capacity. Panel B: Behavioral elicitation methods of risk preference and working memory capacity. Panel C: Self-reported elicitation methods of risk preference and numeracy. Panel D: Behavioral elicitation methods of risk preference and numeracy. Dot shading represents Bayes factors. Error bars indicate 95% frequentist confidence intervals. BF = Bayes factor as evidence for a correlation (H1) over evidence against a correlation (H0); WMC = working memory capacity. See Table 3 for abbreviations of the methods.

Another way to measure response errors is to calculate the absolute difference between responses at two measurement time points in the reliability subsample in Data F. Overall, there was no significant correlation between working memory capacity/numeracy and the absolute difference of answers in the self-report or the behavioral measures (see Fig. 4). Moreover, in more than half of the individual measures, we found Bayesian support for a null-correlation between these two variables. Thus, the idea that participants with higher working memory capacity/numeracy gave fewer erroneous responses across the two measurement points was not supported.

### Robustness

To examine the robustness of our results, we took the residuals of a regression with the predictors age, sex, socioeconomic status (only in Data F), income, and education on each individual risk preference measure. We then repeated the main analyses with the residuals. The qualitative results remained very similar. In particular, there was an overall positive correlation between working memory capacity and risk tolerance for self-report measures, however, this effect was not significant for numeracy anymore. As before no overall significant correlations emerged between working memory capacity/numeracy and the behavioral measures of risk preference. Finally, there remained a positive correlation between working memory capacity/numeracy and EV maximizing for the behavioral methods. All results can be seen in Supplemental Figures S1—S3.

In addition, for the lottery task and the multiple price lists, we estimated random utility models that are frequently used to characterize latent risk preference in these tasks. Because of the nonlinear relation between choice proportions and latent risk preference parameters, it could be that correlations change^59^. However, consistent with the above results, there was no clear Bayesian evidence for a correlation between working memory capacity/numeracy and latent risk preference (see Supplemental Information). in the Supplemental Information we further provide preliminary evidence for the influence of decisions from description versus experience and option complexity on the working memory capacity/numeracy–risk preference relation.

## Discussion

How do working memory capacity and numeracy, facets of cognitive abilities, relate to risk preference? We found that this link is contingent on the elicitation method for risk preference. We observed a small but significant average correlation of around 0.05–0.10 for self-report measures. However, the magnitude of this correlation was smaller than some researchers previously reported^25,60^. As past results were mostly based on only a small subset of the elicitation methods examined here, our findings are more representative for the set of existing elicitation methods of risk preference. In contrast to the self-report measures, we found no significant average correlation between risk preference elicited in behavioral methods and working memory capacity/numeracy.

There was considerable heterogeneity in the correlations between working memory capacity/numeracy and risk preference within the class of behavioral tasks. We attribute this to systematic differences in the choice architecture of the behavioral tasks. Behavioral tasks designed to elicit risk preferences create their own environment through supposedly incidental design features of the choice architecture. One is whether, in a given choice architecture, the strategy of EV maximizing is tantamount to taking risks. This matters for research on the cognitive abilities–risk preference relation because we found a significant correlation between working memory capacity/numeracy and EV-maximizing behavior^61^. In choice architectures where the risky option is also the EV-maximizing option, a spurious link between cognitive abilities and seeming risk preference can emerge. This has severe consequences for external validity, as the observations say little about how people would decide in a choice architecture where risk is not rewarded. Furthermore, such a confound in the choice architecture can explain positive as well as negative correlations between cognitive abilities and risk tolerance.

Our results are generally in line with a recent meta-analysis focusing on binary choices in the class of behavioral measures that found a small positive correlation between cognitive abilities and risk tolerance in the gain domain, but no correlation in the mixed and loss domains^38^. Additionally, this meta-analysis did not find strong moderators of this relation, neither in personal characteristics nor in features of the risk preference elicitation method. However, this study did not code for the choice architecture in terms of the relation between risk taking and rewards in the different experiments. Yet, even then it might be difficult to find effects of the choice architecture across experiments, where participant pools and other task features differ, and which were not always high powered in terms of sample size. Therefore, we think it is worthwhile to examine a multi-elicitation method approach as adopted in the present work. A complementary approach would be to systematically vary all features that could influence the relation between cognitive abilities and risk preference. In particular, future experiments could try to vary the risk–reward relation within a task environment systematically to examine how participants adapt to these changes, and whether cognitive abilities are related to this adaptation.

We also examined how working memory capacity/numeracy relates to response errors. Response errors are another way that choice architectures can affect measures of risk preference. Response error is difficult to observe and usually requires estimating a formal model that includes a quantitative error theory (e.g.,^50,62^). For the subset of methods for which we were able to estimate such models, we found that people high in working memory capacity/numeracy chose more consistently, or made fewer choice errors, in line with previous findings^39,52–54^. Consequently, the impact of response error must also be carefully controlled for when implementing a behavioral elicitation method. Finally, for a subset of participants who responded to each elicitation method twice, we found no significant correlation between working memory capacity/numeracy and the reliability between the two measurement points. This indicates that people with lower cognitive abilities do not show lower temporal stability in their choices. Thus, latent choice consistency and temporal reliability appear to be different constructs. The former might be associated with attention and working memory, whereas the latter might be more closely associated with the malleability of preferences over time or long-term memory processes.

One potential limitation of our study is that the participant pools consisted of a sample of students in a narrow age range^37^ and the general U.S. population^57^. The convergent results in both data sets might speaks against a large effect of the participant pool, but it is nevertheless possible that stronger positive correlations between cognitive abilities and risk tolerance could be found in more extreme samples (extreme groups in age, income, or cognitive abilities; e.g.,^63^). Further, working memory capacity was measured differently in the two data sets. There is some debate in the literature about how to measure working memory capacity and how to distinguish it from related concepts^64,65^. By using different measures of working memory capacity, we believe our results are representative for the elicitation methods used in the field. Caution must be taken when trying to interpret the link between working memory capacity/numeracy and any of our measures of risk preference causally. In particular, as working memory capacity might be correlated with numeracy as well as with other cognitive abilities, reported correlations could turn out to be smaller by adding other psychological constructs with shared variance. Eventually, only precise theories of risk preference and its relation to other psychological constructs can increase trust in causal interpretations. Finally, as working memory capacity and numeracy are only two facets of the larger construct of cognitive abilities, future studies are necessary to confirm the impact of risk preference elicitation methods on the cognitive abilities–risk preference link for other measures of cognitive abilities.

Our results highlight the importance of carefully selecting and designing elicitation methods to measure risk preferences. Choice architectures—especially in behavioral measures—can affect whether correlations between cognitive abilities and risk preference are found, as well as the nature of those correlations. Generalizing from a behavior observed through the lens of a single risk preference elicitation method to a real-world context is only possible if one also controls for all the properties of the choice architecture that can influence risk preference as it is being measured^66^; otherwise the risk of drawing erroneous conclusions from behavioral measures is real. In contrast, self-report measures do not construct their own choice architecture but rather draw on diagnostic past experiences (in existing choice architectures). To the extent that self-report measures address behavior in the domain under examination, they should have a higher validity with respect to real-world behavior^37^. Behavioral and self-report measures might also differ in the kind of preferences they elicit. Behavioral measures may address situational preferences when attention and performance are relatively high, whereas self-report measures are likely to address more stable and abstract attitudes derived from previous decisions with average performance^67^. Moreover, behavioral measures, which are popular for a particular experimental effect, often have low between-subjects heterogeneity and hence low reliability^68^. This makes it harder to find correlations to other psychological constructs for these behavioral measures compared to self-report measures that often have a higher reliability. Thus, when investigating an important question such as whether there is a link between cognitive abilities and risk preference, a thorough analysis should be based on multiple elicitation tools, because using just a single elicitation method risks to lead to unrepresentative results.

Coming back to the relation between cognitive abilities and stock market participation, our results suggest that this relation is more likely a result of EV-maximizing strategies rather than increased risk tolerance. More generally, the competing hypothesis of a positive link between cognitive abilities and EV maximizing should receive more attention in future research. This hypothesis is also compatible with the reported positive relation between cognitive abilities and risk tolerance in self-report measures; this relation could reflect a real-world choice architecture in which those riskier actions we consider are oft accompanied by higher average rewards. Yet, the effect sizes of these general correlations in our two large-scale multimethod data sets were smaller than previously reported^25,60^, which indicates a rather small general and systematic impact of individual differences in cognitive abilities on decisions under risk^69^. From the perspective of research methods, we conclude that risk preference and its effect on behavior must be studied in the context of a carefully constructed and sufficiently understood choice architecture in the laboratory or beyond to generate generalizable results.

## Method

We used two existing data sets that both had a large variety of risk preference elicitation methods and that estimated working memory capacity. For an overview of all experimental tasks, see Table 3. The study producing Data F was approved by the Basel Ethics Committee and the Ethics Committee of the Max Planck Institute for Human Development, Berlin. The study producing Data E was approved by the Stanford Institutional Review Board (IRB-34926). For both experiments informed consent from all participants was collected and all methods were carried out in accordance with relevant guidelines and regulations. For a detailed explanation of all methods as well as how the experimental data were collected, see Frey et al.^37^ for Data F and Eisenberg et al.^57^ for Data E. In the following we describe which data we used and how we analyzed it. As a measure of risk preference, we used either the averaged or summed values of Likert scale answers in self-report questionnaires or the average number of risky choices in the behavioral tasks; for both measures, higher values indicate higher risk tolerance. We always report Pearson correlation coefficients.

Frey et al.^37^ had a homogeneous sample of 1,507 students in the age range of 18 to 34 years (*M* = 25, *Med* = 25, *SD* = 3; 934 female, 573 male) from the Basel–Berlin Risk Study. The data set contains measures of 36 methods for eliciting risk preferences (see Table 3). As a measure of working memory capacity, we used a factor score from four working memory capacity tasks, namely, Memory Updating, Sentence Span, Operation Span, and Spatial Short Term Memory^70,71^. This factor score had *M* = 0.00, *Med* = 0.06, and *SD* = 0.85. As a measure of numeracy^22^, the ability to calculate with probabilities and other numbers, we used the number of correct answers from four questions. Participants correctly solved *M* = 1.76, *Med* = 2 questions, and the dispersion was *SD* = 1.20. In addition, we used a subsample of 171 participants who answered all risk preference measures twice within 6 months to examine temporal stability of the elicited measures. For the descriptive statistics of all examined variables in this data set see Table 4. For the correlation matrix between all measures of working memory capacity, its latent factor, and numeracy see Tables 5.

**Table 4:** Descriptive statistics of variables in Data F.

	Min	Max	Mean	Median	SD	Skew	Kurtosis	Reliability
MUpc	0	1	0.55	0.56	0.21	− 0.31	2.74	0.81
OSpan	0.04	1	0.76	0.78	0.14	− 1.54	7.01	0.7
SSpan	0	0.99	0.68	0.7	0.16	− 1	4.42	0.77
SSTM	0.59	1.11	0.83	0.83	0.08	0	2.95	0.34
WMC	− 3.29	1.85	0	0.06	0.85	− 0.44	3.1	0.85
Numeracy	0	4	1.76	2	1.2	0.13	2.09	0.64
SOEP	1	11	6.08	6	1.93	− 0.1	2.04	0.67
SOEP_dri_	1	11	3.81	3	2.31	0.58	2.41	0.73
SOEP_fin_	1	11	3.69	3	2.07	0.69	3	0.57
SOEP_rec_	1	11	6.7	7	2.16	− 0.56	2.81	0.64
SOEP_occ_	1	11	6.08	6	2.13	− 0.25	2.61	0.53
SOEP_hea_	1	11	5.17	5	2.57	0.06	1.98	0.66
SOEP_soc_	1	11	5.81	6	2.53	− 0.11	2	0.62
D_eth_	1	4.38	1.99	1.88	0.69	0.75	3.17	0.8
D_inv/fin_	1	4.75	1.74	1.5	0.8	0.95	3.03	0.71
D_gam_	1	4.75	1.33	1	0.56	2.33	9.42	0.72
D_hea_	1	4.75	2.64	2.62	0.68	0.23	2.72	0.81
D_rec_	1	5	2.63	2.62	0.87	0.15	2.25	0.83
D_soc_	1	4.88	3.4	3.38	0.55	− 0.26	3.27	0.64
GABS	0	1	0.46	0	0.5	0.16	1.03	0.6
SSSV	41	79	62.95	63	6.1	− 0.23	2.69	0.84
PRI	0	8	4.32	4	1.48	− 0.1	2.76	0.67
BIS	39	102	65.18	65	9.78	0.17	2.85	0.83
Lottery	0.18	0.82	0.39	0.38	0.09	1.06	5.17	0.08
MPL	0	1	0.53	0.54	0.17	− 0.13	3.13	0.44
CCT-cold	0	1	0.23	0.21	0.12	1.19	6.18	0.62
MT	0.6	0.84	0.74	0.74	0.02	− 0.53	5.75	0.47
Traffic	2.05	14.43	8.24	8.22	1.46	0.07	3.41	0.76
BART	2.18	89	40.48	40.57	12.3	− 0.02	2.89	0.55
DfE	0	1	0.5	0.5	0.19	0.11	3.42	0.06
DfD	0	1	0.48	0.5	0.18	0.1	2.97	0.27

MU = Percentage correct in memory updating task; OSpan = percentage correct in operation span task; SSpan = percentage correct in sentence span task; SSTM = similarity score in short term memory task; WMC = factor score of all four working memory tasks (measure used in all reported correlations between working memory capacity and risk preference for Data F); numeracy = score of correctly answered multiple choice questions from 0 to 4. For all other abbreviations see Table 3. Summary statistics are based on 1,507 participants; reliability is based on the Pearson correlation of a subset of 171 participants.

**Table 5:** Correlations between working memory capacity and numeracy measures for Data F.

	MU	OSpan	SSpan	SSTM	WMC
OSpan	0.55	1	—	—	—
SSpan	0.56	0.76	1	—	—
SSTM	0.30	0.20	0.23	1	—
WMC	0.92	0.77	0.80	0.43	1
Numeracy	0.45	0.31	0.27	0.16	0.43

For abbreviations of the measures see Table 4.

Eisenberg et al.^57^ used a more heterogeneous participant pool of 522 participants in the age range of 20 to 59 (*M* = 34, *Med* = 32, *SD* = 8; 260 female, 262 male) recruited through Amazon Mechanical Turk. From their elicited measures, we used 12 that are related to the construct of risk preference. As a measure of working memory capacity we elicited a latent working memory capacity factor derived from a confirmatory factor analysis with one factor based on the scores of five tasks: a digit span task, where participants have to report digits that are presented sequentially in the correct order; a reversed digit span task; a regular and a reversed spatial span task; and an *n*-back task (*M* = 0.00, *Med* = 0.00, *SD* = 0.15). For this data set descriptive statistics of all examined variables are reported in Table 6 and the correlation matrix for measures of working memory capacity is presented in Table 7.

**Table 6:** Descriptive statistics of variables in Data E.

	Min	Max	Mean	Median	SD	Skew	Kurtosis
N-Back	0.05	1.58	0.69	0.67	0.3	0.22	2.98
SSpan	3.3	8.5	5.93	5.9	0.85	− 0.24	3.23
SSpan-rev	3	8.3	5.55	5.5	0.88	0.22	3.12
DSpan	4.7	11.1	7.75	7.8	1.09	0.12	3.12
DSpan-rev	2.4	11	6.69	6.7	1.42	0.07	3.27
WMC	− 0.51	0.39	0	0	0.15	− 0.18	3.13
D_eth_	1	4.83	2.2	2	0.84	0.53	2.61
D_inv/fin_	1	6.17	2.99	2.83	1.07	0.35	2.86
D_hea_	0	1.54	0.42	0.41	0.33	0.51	2.54
D_rec_	1	6.5	2.89	2.83	1.15	0.51	2.89
D_soc_	1.17	6.67	3.89	4	0.85	− 0.12	3.2
BIS	5.14	14.36	9.07	9	1.83	0.34	2.77
SSSV	0	3.69	1.59	1.54	0.75	0.4	2.67
CCT-hot	1	30.1	12.72	11.96	6.08	0.58	2.85
CCT-cold	0.92	26.38	11.03	10.56	4.85	0.52	2.97
H&L	0	10	6.7	7	2.12	− 0.4	2.69
Angling-k	0	61.92	30.88	31.1	11.67	− 0.15	2.66
Angling-r	0	50	21.68	21.44	8.9	0.2	2.89

N-back = Percentage correct button presses in an *n*-back task; SSpan = percentage correct in spatial span task; SSpan-rev = percentage correct in a reversed spatial span task; DSpan = percentage correct in digit span task; DSpan-rev = percentage correct in a reversed digit span task; WMC = factor score of all five working memory tasks (measure used in all reported correlations between working memory capacity and risk preference for Data E). For all other abbreviations see Table 3.

**Table 7:** Correlations between working memory capacity and numeracy measures for Data E.

	N-Back	SSpan	SSpan-rev	DSpan	DSpan-rev
SSpan	0.36	1	—	—	—
Sspan-rev	038	0.58	1	—	—
DSpan	0.28	0.19	0.21	1	—
DSpan-rev	0.35	0.25	0.28	0.51	1
WMC	0.66	0.80	0.83	0.47	0.56

*Note*. For the abbreviations of the measures see Table 6.

### Supplementary Information

Supplementary Information.

## Data Availability

All analyzed data can be retrieved from https://osf.io/rce7g (Data F), https://osf.io/4j9hd, and https://github.com/IanEisenberg/Self_Regulation_Ontology/tree/master/Data/Complete_02-16-2019 (Data E). The code for the analyses presented in this manuscript can be found at https://osf.io/z45ep/.
