# Supplementary material for: The link between cognitive abilities and risk preference depends on measurement
Source: Sci Rep. 2023 Nov 30;13:21151. doi: 10.1038/s41598-023-47844-9 (PMC10689477; doi:10.1038/s41598-023-47844-9)
Supplement: Supplementary file 1 — Supplementary Information. [file 41598_2023_47844_MOESM1_ESM.pdf]

Supplemental Information for:

The Link Between Cognitive Abilities and Risk Preference Depends on Measurement

Sebastian Olschewski[1,2], Jörg Rieskamp[1,+], & Ralph Hertwig[3,+]

[1] Department of Psychology, University of Basel

[2] Warwick Business School, University of Warwick

[3] Max-Planck Institute of Human Development, Berlin

[+] Equal Contribution

Correspondence regarding this SI should be sent to: Sebastian Olschewski, Center for Economic Psychology, Missionsstrasse 62A, 4056 Basel, Switzerland. Email: sebastian.olschewski@unibas.ch

Additional Analyses: Working Memory Capacity and other Characteristics of Decision Making

We verified that our working memory capacity (WMC) measure is meaningfully correlated with other measures of cognitive performance. Indeed, WMC was correlated with the number of samples drawn in the decisions-from-experience task ($r = .24$; Frey et al., 2017). This is consistent with past findings (Frey et al., 2015; Rakow et al., 2008, 2010). Second, WMC was negatively correlated with the propensity of making a logical mistake in the multiple-price list task (MPLs: $r = .–24$ in Frey et al., 2017; and $r = –.10$ in Eisenberg et al., 2019). Again, this finding is expected conceptually and echoes past empirical results (Burks et al., 2009; Mechera-Ostrovsky et al., 2022). In addition, these correlations also suggest that both data sets involved enough variability in individuals' WMC to detect meaningful correlations.

Additional Analyses: The Choice Architecture of Behavioral Elicitation Methods

The choice architectures of the behavioral methods represent either experience-based or description-based choice. In the former, information is encountered or searched sequentially and needs to be integrated; in the latter all information is readily summarized. CCT, Bart, DfE, and the angling task are experience-based, whereas lotteries, MPL, and DfD are description-based. The pattern of correlations within the experience-based category is very heterogeneous with significant positive, significant negative and almost zero correlations. In contrast, within all description-based measures there was no evidence for a significant correlation between risk preference and WMC. In sum, across description and experienced-based behavioral elicitation tasks there is no consistent link between WMC and risk preference. However, the stronger absolute correlations in the experience-based tasks could hint to the hypothesis that WMC has a stronger influence on risk taking in environments where information has to be searched and integrated, rather than when all information is readily presented.

A choice architecture could also present two options that differ in their cognitive (processing) demand. If, for example, the task is to choose between a sure outcome and a risky lottery or between risky lotteries with varying numbers of outcomes, differences in the number of processing steps could make people perceive these options with different degrees of precision (Olschewski & Rieskamp, 2021). People with higher cognitive ability might be better able to process the numeric information of more complex options more precisely and are thus willing to choose them more readily (Burks et al., 2009; Oberholzer et al., 2024;

Zilker et al., 2020). To the extent that the complexity of options is not controlled for in a choice architecture, this is another way to cause a spurious correlation between higher cognitive abilities and risk preference.

An objective measure of complexity is, for example, the number of outcomes a choice option offers. In the examined data, this was controlled for when designing most tasks. For example, in the lottery and the MPL tasks, there were only choice options with two risky outcomes. However, in the DFD choice set in Frey et al. (2017), there were four choices (two in the gain and two in the loss domain), where the choice was between one risky two-outcome lottery and a sure single outcome. When correlating WMC with the average of risky choices in this subset, there is a significant positive correlation, $r = .05$, $p = .004$, thus supporting the finding in Burks et al. (2009) that participants with higher cognitive ability are more willing to choose more complex choice options.

Partial Correlation Between Working Memory Capacity/ Numeracy and Risk Preference

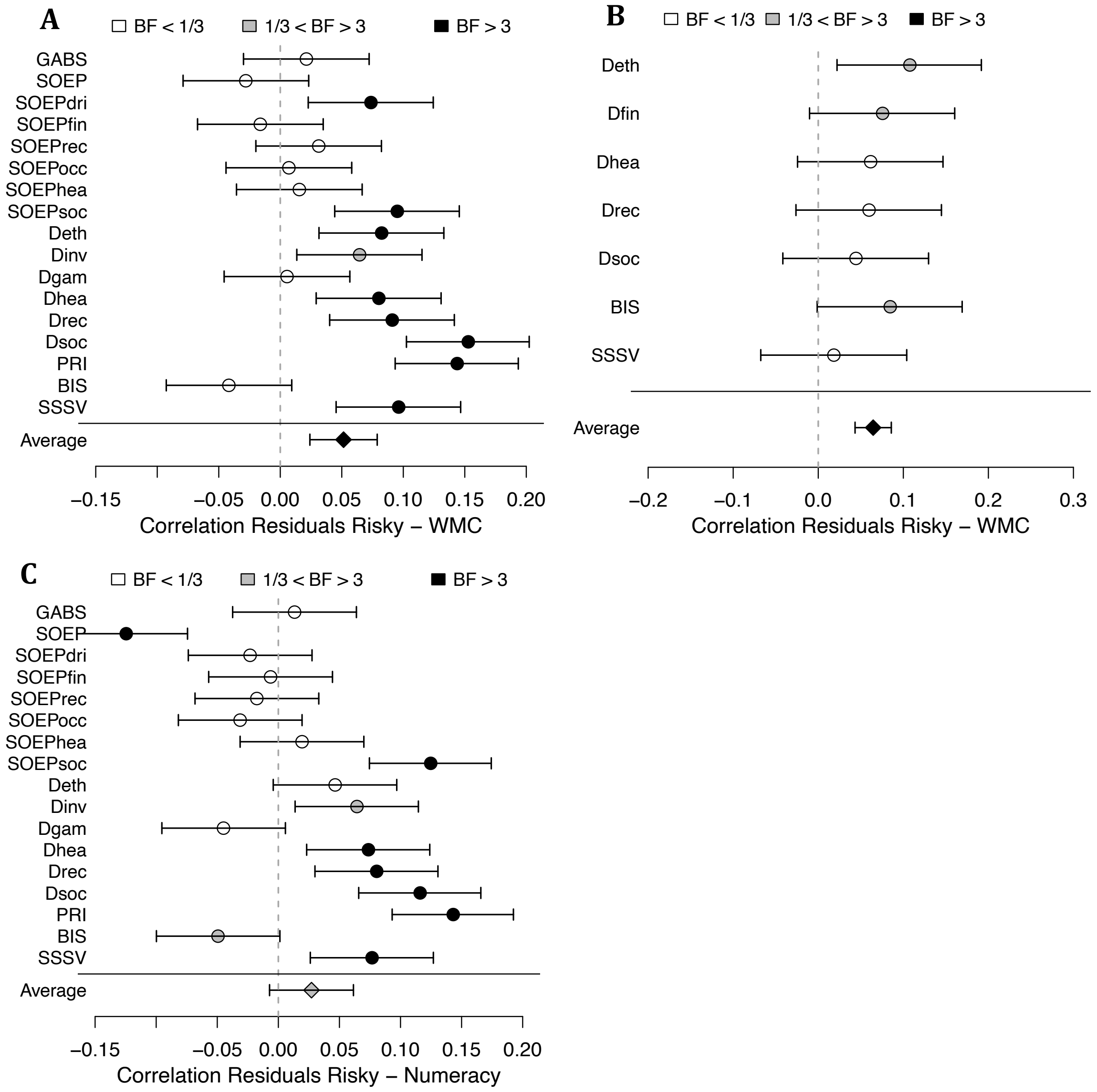

Figure S1. Self-report risk preference elicitation methods and their partial correlation with working memory capacity (WMC) and numeracy. Correlations with residuals from a regression with predictors: age, sex, socio-economic status (only available in Frey et al., 2017), income, and education. Panel A: WMC and self-report risk preference residuals in the data of Frey et al. (2017). Panel B: WMC and self-report risk preference residuals in the data of Eisenberg et al. (2019). Panel C: Numeracy and self-report risk preference residuals in the data of Frey et al. (2017). Error bars are 95% frequentist confidence intervals. BF = Bayes factor as evidence for a correlation (H1) over evidence against a correlation (H0). See Table 3 in the main text for abbreviations of the methods.

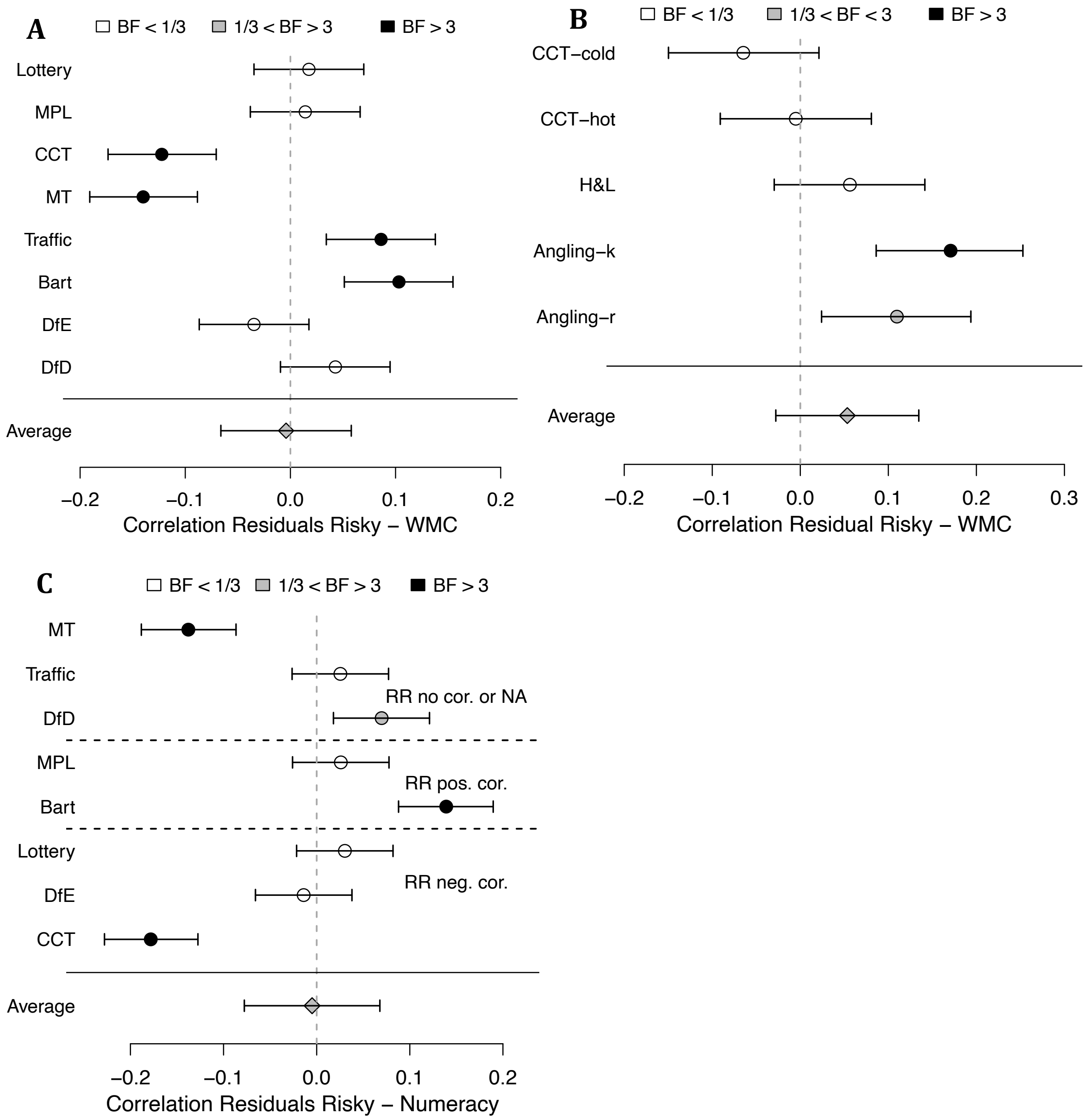

Figure S2. Behavioral risk preference elicitation methods and their partial correlation with working memory capacity (WMC) and numeracy. Correlations with residuals from a regression with predictors: age, sex, socio-economic status (only in Frey et al., 2017), income, and education. Correlations with residuals from a regression with predictors: age, sex, socio-economic status (only available in Frey et al., 2017), income, and education. Panel A: WMC and behavioral risk preference residuals in the data of Frey et al. (2017). Panel B: WMC and behavioral risk preference residuals in the data of Eisenberg et al. (2019). Panel C: Numeracy and behavioral risk preference residuals in the data of Frey et al. (2017). Error bars are 95% frequentist confidence intervals. BF = Bayes factor as evidence for a correlation (H1) over evidence against a correlation (H0). See Table 3 in the main text for abbreviations of the methods.

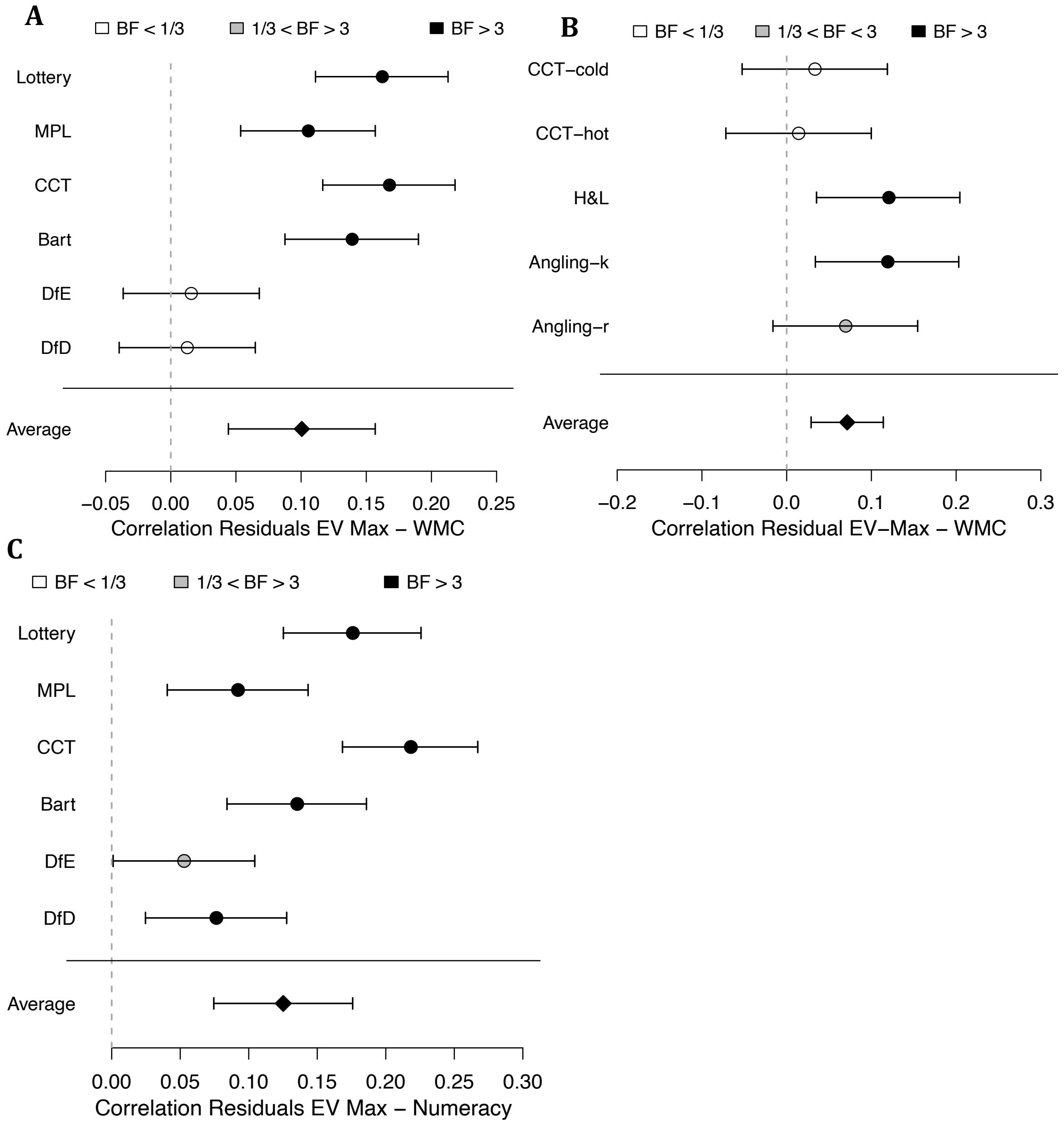

Figure S3. Behavioral elicitation methods allowing for the calculation of EV-maximizing behavior and their partial correlation with working memory capacity (WMC) and numeracy. Correlations with residuals from a regression with predictors: age, sex, socio-economic status (only in Frey et al., 2017), income, and education. Panel A: WMC and behavioral EV-maximizing residuals in the data of Frey et al. (2017). Panel B: WMC and behavioral EV-maximizing residuals in the data of Eisenberg et al. (2019). Panel C: Numeracy and behavioral EV-maximizing residuals in the data of Frey et al. (2017). Error bars are 95% frequentist confidence intervals. BF = Bayes factor as evidence for a correlation (H1) over evidence against a correlation (H0). See Table 3 in the main text for abbreviations of the methods.

Behavioral Risk Preference Measures and Latent Variable Correlations

Using a Bayesian hierarchical model estimated in JAGS, we estimated a random utility model with probit link function to capture decision error with θ representing choice consistency (see main text), and a mean-variance function with β to capture latent risk preference:

$U_x = EV_x + \beta \times SD_x$,

$U_y = EV_y + \beta \times SD_y$,

$p(y|\{x, y\}) = \Phi\left(\frac{U_y - U_x}{\theta}\right)$.

We applied this model to the lottery task and the MPLs. These tasks are frequently described with random utility models and have a repeated measurement design to allow for sensible estimates. Other tasks are less suitable for estimating random utility models. This is the case because tasks like Bart, Angling and CCT have trials that are not independent, tasks like MT and VT have no variability in EV, and tasks like DfD and DfE have too few choices per participant.

As results, we replicated the low correlations between WMC and behavioral risk preference measures in the lottery and the MPL task in Frey et al. (2017) as well as in the Holt & Laury price list in Eisenberg et al. (2019), also when using latent risk preference measures: $r(\beta_{\text{Lottery}}, WMC) = 0.04$, $p = .053$, $BF_{1,0} = 0.19$; $r(\beta_{\text{Lottery}}, \text{Numeracy}) = 0.06$, $p = .012$, $BF_{1,0} = 1.39$; $r(\beta_{\text{MPL}}, WMC) = 0.03$, $p = .231$, $BF_{1,0} = 0.12$; $r(\beta_{\text{MPL}}, \text{Numeracy}) = 0.06$, $p = .058$, $BF_{1,0} = 0.36$); $r(\beta_{\text{HL}}, WMC) = 0.10$, $p = .026$, $BF_{1,0} = 1.19$.

Supplemental References

Burks, S. V., Carpenter, J. P., Goette, L., & Rustichini, A. (2009). Cognitive skills affect economic preferences, strategic behavior, and job attachment. *Proceedings of the National Academy of Sciences*, *106*(19), 7745–7750. https://doi.org/10.1073/pnas.0812360106

Eisenberg, I. W., Bissett, P. G., Zeynep Enkavi, A., Li, J., MacKinnon, D. P., Marsch, L. A., & Poldrack, R. A. (2019). Uncovering the structure of self-regulation through data-driven ontology discovery. *Nature Communications*, *10*(1), 1–13.

Frey, R., Mata, R., & Hertwig, R. (2015). The role of cognitive abilities in decisions from experience: Age differences emerge as a function of choice set size. *Cognition*, *142*, 60–80. https://doi.org/10.1016/j.cognition.2015.05.004

Frey, R., Pedroni, A., Mata, R., Rieskamp, J., & Hertwig, R. (2017). Risk preference shares the psychometric structure of major psychological traits. *Science Advances*, *3*(10), e1701381. https://doi.org/10.1126/sciadv.1701381

Mechera-Ostrovsky, T., Heinke, S., Andraszewicz, S., & Rieskamp, J. (2022). Cognitive abilities affect decision errors but not risk preferences: A meta-analysis. *Psychonomic Bulletin & Review*, 1–32.

Oberholzer, Y., Olschewski, S., & Scheibehenne, B. (2024). Complexity aversion in risky choices and valuations: Moderators and possible causes. *Journal of Economic Psychology*, 100, https://doi.org/10.1016/j.joep.2023.102681.

Olschewski, S., & Rieskamp, J. (2021). Distinguishing three effects of time pressure on risk taking: Choice consistency, risk preference, and strategy selection. *Journal of Behavioral Decision Making*, *34*(4), 541–554.

Rakow, T., Demes, K. A., & Newell, B. R. (2008). Biased samples not mode of presentation: Re-examining the apparent underweighting of rare events in experience-based choice. *Organizational Behavior and Human Decision Processes*, *106*(2), 168–179. https://doi.org/10.1016/j.obhdp.2008.02.001

Rakow, T., Newell, B. R., & Zougkou, K. (2010). The Role of Working Memory in Information Acquisition and Decision Making: Lessons from the Binary Prediction Task. *Quarterly Journal of Experimental Psychology*, *63*(7), 1335–1360. https://doi.org/10.1080/17470210903357945

Zilker, V., Hertwig, R., & Pachur, T. (2020). Age differences in risk attitude are shaped by option complexity. *Journal of Experimental Psychology: General*, *149*(9), 1644.
